# Conservation of Native Livestock Breeds in Russia: Current State and Promising Prospects

**DOI:** 10.3390/ani15213103

**Published:** 2025-10-25

**Authors:** Natalia A. Zinovieva, Tatiana E. Deniskova, Veronika R. Kharzinova, Vugar A. Bagirov, Michael N. Romanov, Valeriya V. Volkova, Dinara S. Grishina, Alexandra S. Abdelmanova, Igor V. Gusev, Ivan M. Shchukin, Vladimir I. Trukhachev, Oksana I. Boronetskaya

**Affiliations:** 1 L.K. Ernst Federal Research Center for Animal Husbandry, Dubrovitsy, Podolsk Municipal District, Podolsk 142132, Russia; horarka@yandex.ru (T.E.D.); veronika0784@mail.ru (V.R.K.); preevetic@mail.ru (A.S.A.);; 2 Upper Volga Federal Agricultural Scientific Center, Novy, Suzdal District, Suzdal 601261, Russia; 3 Russian State Agrarian University–Moscow Timiryazev Agricultural Academy, Moscow 127434, Russia

**Keywords:** livestock, animal genetic resources, genetic diversity, genetic monitoring, native breeds, ex situ conservation, in situ conservation, in vitro technologies, in vivo biocollections, molecular genetic techniques

## Abstract

**Simple Summary:**

Global intensification of livestock industry, despite its growing output, has negative consequences. In particular, there has been a sharp reduction in the number and, consequently, the genetic diversity of animal genetic resources (AnGR). The use of several specialized breeds, the genetic diversity of which is also reduced due to inbreeding and targeted intensive selection, can damage food security, especially in the event of unforeseen climate change and epidemics. Therefore, the development and implementation of scientifically based strategies for the conservation of genetic material from animals of native breeds and from outstanding representatives of commercial breeds is an important task for national governments. In Russia, the National Center for AnGR was established, and bioresource collections of farm animals are maintained on a competitive grant basis. Successful preservation case examples include the Tagil and Kostroma cattle breeds and the Orenburg goat breed, alongside genetic standards for native breeds created using museum specimens. Research is also being conducted in Russia to develop conservation strategies for AnGR, particularly for breeds that are in *at risk*, *vulnerable*, *endangered* and *critical* status. The potential to preserve the genetic diversity of native AnGR for future generations is encouraged by successful instances of these national programs being implemented.

**Abstract:**

This review summarizes and analyzes the current status and trends in conservation of agricultural AnGR in Russia. The biodiversity of farm animal species in Russia is significant and is represented by 507 breeds, including 236 aboriginal ones. Based on a summary of global experience in maintaining genetic material of various types, we describe here strategies for preserving native breeds in Russia. Genetic monitoring using modern technologies improves the relevance of biological collections and enables the selection of the most typical and valuable representatives of AnGR for cryopreservation. The National Center for AnGR has developed a key conservation strategy based on the integrated use of genetic and assisted reproductive technologies. This strategy has been successfully implemented in a few cattle and goat breeds. In 2025, genetic monitoring of AnGR reproduced in vivo and preserved in vitro began. These studies and practical measures in Russia play an important role in preserving the genetic diversity of native AnGR in a changing climate to ensure food security for current and future generations. Thus, owing to the joint efforts of research teams and state financial support, a positive trend has emerged in cryopreservation programs and the preservation of living bioresource collections in Russia.

## 1. Introduction

Animal genetic resources (AnGR) are among the most valuable and strategic resources in every country, providing 18% of total calories and 39% of protein intake in human nutrition [1]. The dramatic decline in global biodiversity has been observed in recent decades, both in Russia and worldwide, due to the orientation of agricultural production systems toward the use of a limited number of breeds and the resultant reduction in the number of local genetic resources. This creates risks of lowering the sustainability of agricultural production systems and their ability to adapt to changeable environmental conditions (i.e., alterations in technogenic load and climate change) and market needs (e.g., creation of geographically oriented production systems and production of organic products) [2,3,4,5]. In addition, valuable genotypes carried by native animal breeds may be irretrievably lost [6,7,8]. This determines the high relevance of activities aimed at preserving biodiversity, especially national genetic resources of farm animals.

The increased international concern about the increasing risk of extinction of a large number of species resulted in the adoption of the United Nations (UN) Convention on Biological Diversity [9] in 1992 that was the first document aimed at a comprehensive solution to the problem of biodiversity loss (the Russian Federation ratified the Convention by the Federal Law No. 16 of 17 February 1995). The scope of the Convention is the conservation of the diversity of all forms of life, including AnGR. The Convention established the obligations of member countries, including Russia, to ensure sufficient conditions for the conservation of biological diversity, both in the natural habitat of species populations, i.e., in situ (Article 8), and outside the original habitats of species populations, i.e., ex situ (Article 9).

The Food and Agriculture Organization (FAO) of the UN plays a decisive role in developing approaches to the conservation, study and rational use of the biodiversity of AnGR used for food and agriculture at the global level [10]. Conservation is one of the four Strategic Priority Areas of the Global Plan of Action for AnGR developed by FAO [11]. According to FAO [12], *“AnGR encompass the individuals and populations within these species, as well as genetic material (semen, oocytes, embryos,* etc.) *which may exist outside the living animals”.* Aquatic species (wild and farmed) and, in some instances, wild terrestrial animals were excluded from the definition of AnGR because of different management and policy challenges for these species. In this review, we will follow the FAO terminology for AnGR.

As of 1 January 2024, out of a total of 8164 extant breeds (extinct breeds are not taken into account) recorded in the FAO Domestic Animal Diversity Information System (DAD-IS), 7326 breeds were local (including 4927 breeds in mammals and 2134 in birds), 542 were regional transboundary (446 and 96 breeds, respectively) and 561 are global transboundary (402 and 159 breeds, respectively) [13]. According to the population size, four categories were defined to consider breeds in terms of extinction risk, including (1) *at risk*, (2) *vulnerable*, (3) *endangered* and (4) *critical*. Based on expert knowledge, the respective population size thresholds was set on 10,000, 2000, 1000 and 100 animals for the species with *high reproductive capacity* (i.e., those bearing multiple offspring) and 10,000, 6000, 3000 and 300 for the species with *low reproductive capacity* [14,15]. The category *extinct* was introduced to define the breeds that could not be reconstituted [16]. The breeds with no viable in situ populations, but with sufficient material stored in gene banks to allow their reconstitution, were categorized as *cryoconserved only* [15]. It should be noted that expert-driven methods are by far the most commonly used approaches for setting thresholds for conservation management [17]. Additionally the modifier *maintained* was introduced to distinguish populations within the *critical* and *endangered* categories for which active conservation programs were in place [10]. FAO member nations, including Russia, who ratified the Global Plan of Action for AnGR, shall take into account the aforementioned risk categories when developing national policies and action plans on AnGR [11].

In 1989, a comprehensive overview on the extensive AnGR of the former Soviet Union was published as a special FAO Animal Production and Health Paper [18]. The purpose of this review is to provide detailed information, and fill a gap in the knowledge, on the current AnGR status, as well as directions of research work and practical activities in the field of the conservation of genetic resources of agricultural animals in Russia.

## 2. Methodology

This review was compiled based on a thorough search of publicly available reference databases. The search strategy involved using a combination of terms, including “animal genetic resources,” “AnGR,” “gene bank,” “biobanking,” “ex situ,” and “AnGR conservation,” as well as specific text terms corresponding to the above search queries or their variations. The current review focused on peer-reviewed original research articles in English and Russian. To search for articles and other publications, we used databases such as PubMed [19], eLIBRARY.RU ([20]; available in Russian and English), CyberLeninka ([21]; available in Russian and English) and ResearchGate [22]. We also actively conducted a general search using the Google and Google Scholar search engines and analyzed materials from the FAO website [23]. We also used printed archival and periodical scientific materials stored in the Scientific Library of the L.K. Ernst Federal Research Center for Animal Husbandry [24]. In addition, we used materials from oral presentations prepared by employees of the L.K. Ernst Federal Research Center for Animal Husbandry for delivering at national and international scientific meetings and posted on the official website of the L.K. Ernst Federal Research Center for Animal Husbandry [25], as well as scientific reports prepared as part of the implementation of scientific projects and grants.

## 3. Overall Description of the State of the Art of AnGR in Russia

**Figure 1:**
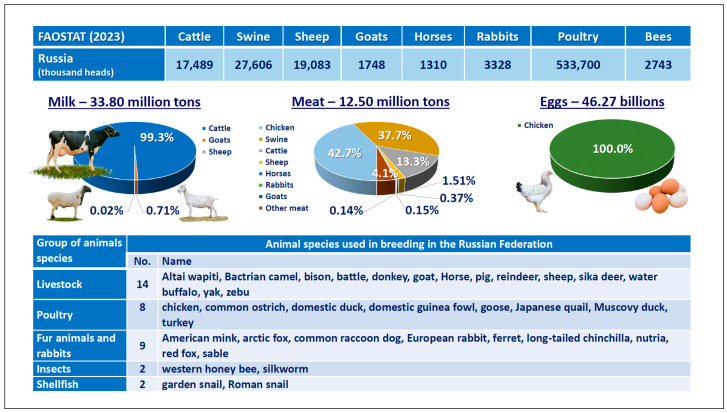
Contribution of farm animals to food security in Russia (according to [26,27,28]) and animal species subject to selective breeding in Russia (according to [26]).

In the Russian Federation, selective breeding is carried out for 35 farm animal species, including 14 livestock species, eight poultry species, nine species of fur animals and rabbits, two insect species and two mollusks species [26]. Meanwhile, four species, including cattle, chickens, pigs and sheep, make a key contribution to ensuring food security. In 2023, milk production was 99.3% provided by one species (cattle), with 95.2% meat produced by four species (chickens, 42.7%; pigs, 37.7%; cattle, 13.3%; and sheep, 1.5%) and 100% eggs produced by chickens [27,28] (Figure 1).

In the Russian Federation, three types of genetic resources are officially recognized and registered, including breeds, intra-breed types and line crosses [29]. In total, 507 breeds have been registered for 35 species of farm animals, including 236 native breeds, 140 intra-breed types and 141 cross lines [26] (Table 1). However, in most cases, livestock production is based on using a limited number of, as a rule, transboundary breeds. For example, the breeding stock of dairy and dairy-beef cattle in 2024 was represented by 24 breeds, including 9 foreign and 15 native ones (Supplementary Table S1) [30]. The total number of breeding stock amounted to 2525.13 thousand animals, including 1552.87 thousand herd-book cows, while 85.8% were the transboundary Holstein breed and the Russian Black Pied breed with a high proportion of inheritance from Holsteins [30]. Fifteen native breeds accounted for 7.5% of the total number of breeding stock, with only five of them having the *not at risk* status [30]. Ten others were at various degrees of risk, including two breeds that had the *at risk* status, one *vulnerable*, four *endangered*, two *critical* and one *cryoconserved only*. In 2022, for the first time since the issue of the stud books (1926), selective breeding of the one of the oldest native breed, the Red Gorbatov [31], was suspended. However, an ex situ population of this breed kept at the L.K. Ernst Federal Research Center for Animal Husbandry is used for the production of in vitro embryos subject to cryoconservation [32]. The Pechora intra-breed type of Kholmogory cattle, the northernmost cattle in the European part of Russia [31], has a critical status (Supplementary Table S1). Another case are dairy goats, in which selective breeding is carried out only among transboundary breeds. In 2024, the total breeding stock of dairy goats was 47.67 thousand animals, of which 85.2% were Saanen, 11.5% were Alpine and the remaining 3.3% were Murciano Granadina, Nubian and Toggenburg breeds (0.4 thousand animals) [33]. Local Russian dairy goat breeds (Russian White, Gorki, Dagestan Dairy and Megrelian) have survived only in private farmsteads. In the latter, selective breeding and recording of the numbers and productive traits are not conducted [33]. A similar situation is observed in other farm animal species, which necessitates the development of measures aimed at preserving native genetic resources.

## 4. Preservation of AnGR in Russia

**Figure 2:**
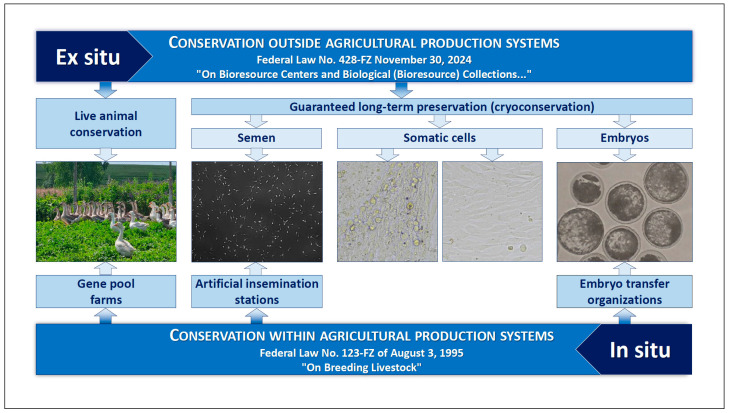
Types of AnGR collections with functionality preservation of units of heredity as recognized in Russia [28].

According to the Russian Federation legislation [34,35], both ways of AnGR conservation are employed in Russia, i.e., both in situ and ex situ preservation, including in vivo (maintenance of live populations of animals) and in vitro (creation of biobanks or cryobanks) approaches [28] (Figure 2).

In Russia, the form of in situ conservation of native agricultural animal breeds are gene pool farms. In addition, bulls of native breeds are kept at artificial insemination (AI) stations of farm animals in order to obtain and cryopreserve semen. Embryos of native breeds are produced at embryo transfer (ET) stations for their subsequent sale to cattle enterprises. However, the inability of native AnGR to compete with transboundary breeds in terms of performance and adaptability to the industrial technology of livestock production leads to variations in the number of breeds and the number of animals preserved in gene pool farms. For example, in 2022–2024, eight native breeds of dairy and dual-purpose cattle with a total number of 3.74–7.14 thousand animals were bred in gene pool farms (Table 2). Due to low demand by farmers, sperm from only a limited number of local breed bulls is preserved at AI stations. Embryo production of local breeds is not practiced due to its commercial unattractiveness. While the maintenance of live animals in gene pool farms is available through government subsidies, financial support for production and cryopreservation of embryos is currently not provided. In general, commercial embryo production in animal husbandry in Russia has not become widespread. In 2022, only 5412 in vitro-derived embryos and 147 in vivo produced embryos were obtained for commercial purposes in Russia [36], accounting for only 1.4% and 0.01%, respectively, of the total volume of cattle embryo production in the world.

It is necessary to point out a number of disadvantages of in situ form. In particular, gene pool farms can be excluded from the register of breeding farms based on the owner’s decision, after which the owners do not bear obligations to conserve genetic resources. In addition, the in situ conservation of AnGR is not focused on preserving biological diversity. The main criterion for selecting animals for in situ conservation is their higher breeding value, i.e., the ability to develop a higher level of economically important traits in the agricultural production system without taking into account allelic diversity. This leads to the use of a limited number of the most productive males, as well as crossing with transboundary breeds, and, as a consequence, to the loss of genetic diversity. Thus, the use of only the in situ measures is not able to fully solve the problem of preserving the biodiversity of AnGR, which necessitates the development of ex situ conservation system.

**Figure 3:**
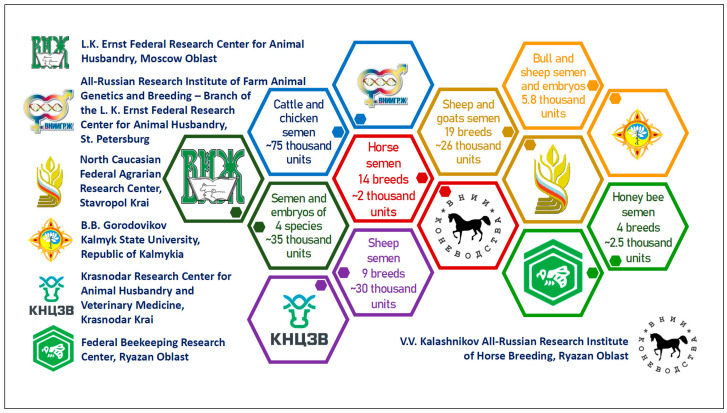
In vitro collections of AnGR germplasm in Russia [28].

The systematic work on the ex situ conservation of AnGR in Russia was launched following the decree of the President of the Russian Federation No. 195 in March 2024 [39]. This called for the establishment of the National Center for Genetic Resources of Agricultural Animals (Russian National Center for AnGR) on the basis of the L.K. Ernst Federal Research Center for Animal Husbandry [28]. The next step was the adoption of the Federal Law No. 428-FZ of 30 November 2024 “On Bioresource Centers and Biological (Bioresource) Collections...” [35]. An inventory of bioresource collections of farm animals conducted by the National Center in 2024 showed that in vivo collections of AnGR were created in six research and educational institutions, where 8160 animals were maintained under live population conservation, including cattle, sheep, goats, horses, chickens, geese, turkeys and silkworm [40]. In vivo conservation is associated with high material costs, so this method is of limited use. The most preferred method for ex situ conservation of AnGR is cryopreservation of germplasm [41,42]. An economic assessment of the effectiveness of cryopreservation measures for Russian AnGR has not been conducted; therefore, internationally recognized estimates [43] can be taken into account when developing AnGR conservation programs in Russia. In vitro collections organized in six research institutions (Figure 3) support 177.3 thousand units of germplasm from 112 breeds of six farm animal species, including cattle, sheep, goat, horse, chicken and honeybee [28,40]. As part of the implementation of the National Center for AnGR development program, it is planned to increase the number of germplasm samples stored in bioresource collections, as well as expand the range of farm animal species and breeds included in in vitro conservation programs [44].

When preserving breeds solely by cryopreserving germplasm, samples from a limited number of the most valuable animals are used, as preserving the entire existing population this way is time-consuming and costly. This limitation particularly applies to embryos and oocytes. This can lead to a decrease in the genetic diversity of breeds. When reconstructing breeds, careful selection of parental pairs will be required to avoid inbreeding depression and other undesirable consequences.

## 5. Conservation and Research of Russian AnGR

The main type of genetic material stored in the in vitro collections of AnGR in Russia is semen. Semen samples from 1081 animals, representing 112 breeds of six animal species (cattle, sheep, goat, horse, chicken and honeybee), account for over 99.6% of the total number of samples. The remaining 0.4% of the samples are bovine embryos, as well as equine embryos and oocytes obtained from a limited number of breeds [40].

### 5.1. Key Conservation Strategy for AnGR

To preserve most of the genetic diversity of Russia’s native breeds at the Russian National Center for AnGR, a key conservation strategy has been developed that is based on the combined use of genetic and assisted reproductive technologies. The strategy includes the following stages [28]:

- Creation of reference DNA profiles of breeds based on molecular genetic studies of archival or historical (museum) samples [45];
- Conducting genetic monitoring of modern populations and creating a resource herd of donor females that have kept the largest proportion of authentic genetic components;
- Producing in vitro and in vivo embryos and placing them in a cryobank for long-term storage;
- Producing offspring from donor females and males selected based on the results of genomic analysis to preserve authentic genomic components in the next generation;
- Replenishment of the resource herd of donor females and sale of males to AI stations;
- Obtaining and cryopreserving semen at AI stations for sale to agricultural enterprises;
- Replenishment of the bioresource collection of the Russian National Center for AnGR with embryo samples from “new” donors and semen samples from males provided by AI stations.

**Figure 4:**
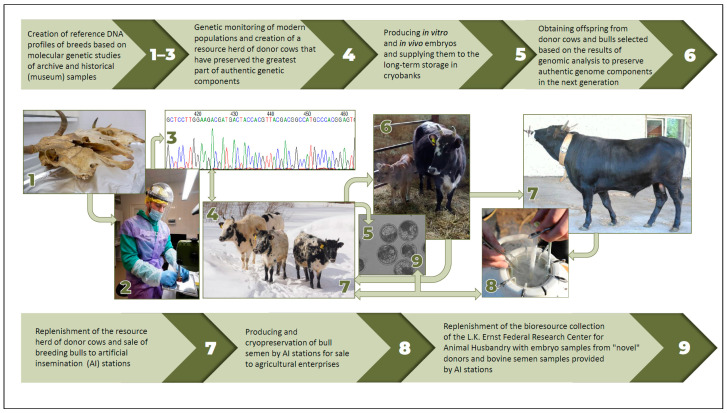
Flowchart representation of the key conservation strategy for genetic resources of cattle breeds based on the combined use of genetic and assisted reproductive technologies as applied at the Russian National Center for AnGR [28].

Schematic representation of the key conservation strategy for AnGR using an example of cattle breeds [28] is presented in Figure 4.

### 5.2. Selection of Animals for Conservation

**Figure 5:**
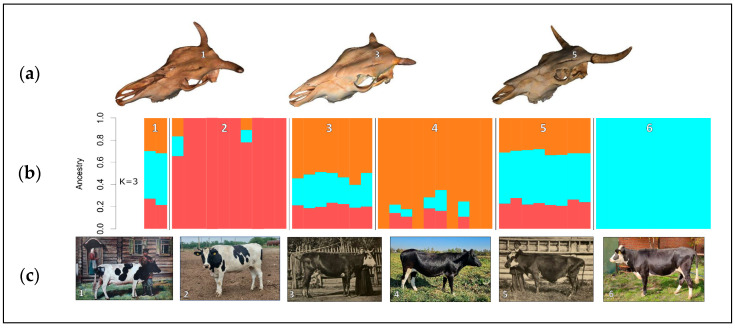
Comparative studies of modern and historical samples of the Russian local cattle breeds based on 255,444 SNPs extracted from WGS data [28]: (**a**) museum samples of cattle sculls (from left to right) of the Great Russian, Kholmogory and Yaroslavl cattle breeds dated back to the end of 19th century to the first half of 20th century and used for DNA recovery and analysis; (**b**) admixture plot using museum samples of the Great Russian (1), Kholmogory (3) and Yaroslavl (5) breeds, along with modern samples of the Holstein (2), Kholmogory (4) and Yaroslavl (6) breeds [31] (the number of clusters K = 3 conforms to the most probable number of ancestors based on CV error calculation); (**c**) pictures (left to right) of historical Great Russian (1), modern Holstein (2; own photo), historical Kholmogory (3; [58]), modern Kholmogory (4; own photo), historical Yaroslavl (5; [58]) and modern Yaroslavl (6; own photo) cattle.

The first stage of the strategy implementation is the selection of samples whose DNA profiles will be used as genetic standards in the subsequent selection of animals for inclusion in conservation programs. This stage is primary to the successful materialization of the entire strategy, since the outcomes of DNA analysis are significantly influenced by the choosing the initial sample pool [2]. Certain subpopulations may later be seen as having questionable legitimacy if their DNA variants are excluded from the breed profile due to their exclusion (intentionally or unintentionally) from the initial analysis. This is particularly crucial in landraces when selection processes, founder effect, isolation, or genetic drift may have created separate subpopulations [2]. Another problem in selecting reference samples for genotyping is that many local breeds have been crossed with transboundary breeds over the past few decades in order to improve productivity and adaptability to industrial technology [46]. Therefore, it is necessary to determine breed-specific genomic components that distinguish local genetic resources from transboundary breeds. Analysis of archival and historical specimens stored in museum collections has great potential for elucidating breed origins and characterizing the genetic architecture of breeds [47,48,49,50]. Studies of ancient and historical DNA in domestic animals can shed light on the history of domestication [49] and the history of local animal husbandry [51,52,53]. For example, the complete genomes of 22 historical honeybee specimens from the collection of the Natural History Museum in Bern were sequenced, some of which were dated back 150 years [54]. The genetic structure of the local Finnish horse breed was studied using historical DNA samples collected before, during and after its creation, covering the period from the end of the 19th century through the 20th century [55]. A comparative molecular genetic study of museum and modern representatives of aboriginal cattle breeds has provided convincing evidence of the preservation of the historical genetic background in modern populations of the Kholmogory and Yaroslavl dairy cattle breeds [56], as well as the Kalmyk, Kyrgyz and Kazakh beef cattle breeds [7]. Thus, molecular genetic studies of museum specimens make it possible to create genetic standards for breeds that will help to trace historical genetic components in modern populations and select the most valuable animals for preservation. Semen samples from bulls born in the 1970s and 1980s that are stored at regional AI stations and in the genetic collection of cattle sperm of native and foreign breeds at the All-Russian Research Institute of Farm Animal Genetics and Breeding–Branch of the L.K. Ernst Federal Research Center for Animal Husbandry can be used as archival samples. The source of historical (museum) samples of the Russian native breeds is the craniological collection of the E.F. Liskun Museum of Animal Husbandry, Russian State Agrarian University–Moscow Timiryazev Agricultural Academy [6,56,57]. For instance, museum skulls of Livny pigs, the only Russian native pig breed adapted to pasture maintenance and green mass feeding, dated back to the 1970s, were used to obtain reference DNA profiles using whole-genome sequencing (WGS). Comparative studies using 52,706 autosomal single nucleotide polymorphisms (SNPs) extracted from WGS data showed the preservation of a significant proportion of native genomic components in modern samples [57]. Museum skull specimens of two of the oldest native cattle breeds, Yaroslavl and Kholmogory [31], dated back to the late 19th and early 20th centuries, were used to trace ancestral genomic components in modern representatives of the breeds (Figure 5a,c). The studies conducted using 255,444 SNPs extracted from WGS data showed the preservation of a significant proportion of ancestral genomic components in modern representatives of the breeds (Figure 5b), as well as the presence of animals that had no visible admixture with Holstein cattle [28]. Animals that carried the majority of ancestral genomic components are priority specimens for use in genetic resource conservation programs.

To produce embryos for the purpose of preserving AnGR, the researchers of the Russian National Center for AnGR mainly utilize in vitro fertilization using OPU oocytes [59]. Introduction of this technology, depending on a breed, allows producing an average of 5.7 to 16.2 embryos per month from each female when employing donors twice a week without the use of hormonal stimulation [60].

### 5.3. Implementation Examples of the Strategy for AnGR Conservation

#### 5.3.1. Tagil Cattle Breed

**Figure 6:**
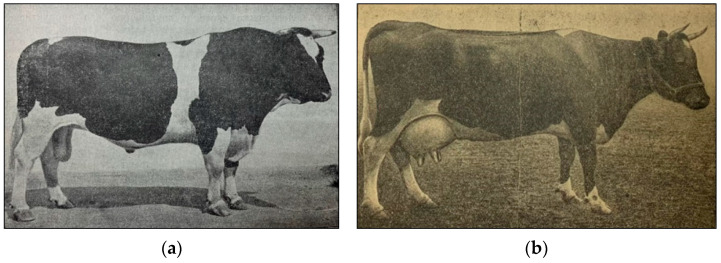

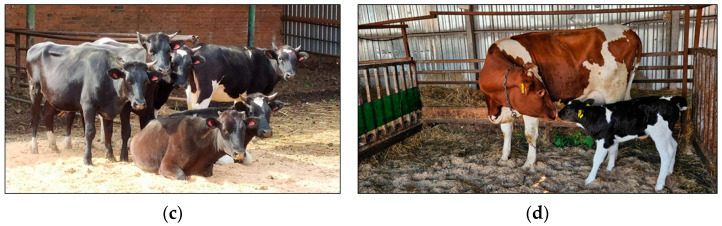
Native Tagil cattle: (**a**) Vampire II UT-68, bull of the Tagil breed (year of birth, 1933; body weight, 1102 kg; breed champion of the All-Union Agricultural Exhibition in 1940); (**b**) Sailor ET-47, cow of the Tagil breed (year of birth, 1933; milk yield for 300 days of lactation, 8082 kg; fat content, 4.28%); (**c**) donor heifers of the Tagil breed (L.K. Ernst Federal Research Center for Animal Husbandry, 2022); (**d**) ET calf of the Tagil breed, along with a recipient cow (L.K. Ernst Federal Research Center for Animal Husbandry, 2022) [28].

There are a number of cases for positive realization of this strategy to reduce the risk status of native breeds. For instance, the Tagil breed, one of the local cattle breeds, has the risk status assessed as *critical*, with its history dated back more than 200 years ago [31,61]. The creation basis for this breed was local low-yielding black pied cattle of the Ural region, and various cattle breeds were used for its improvement at different times. As early as 1873, the Russian scholar Leonid P. Sabaneev [61] wrote that the Tagil breed *“derives from a Kholmogory bull sent as a gift by Peter the Great,”* the Russian Emperor. In the works of the veterinarian Alexei I. Romanov in 1913 [62], it is reported that English short-horned and Kholmogory cattle, brought in 1842 from Arkhangelsk Governorate, participated in the creation of the Tagil breed. Crossbreeding of local cattle with Kholmogory was carried out until 1866. In subsequent periods, the greatest influence on the formation of the allele pool of Tagil cattle was exerted by Yaroslavl and Dutch cattle [63]. According to Alexei I. Romanov [62], *“Tagils are to some extent similar to Kholmogory and Dutch cattle, were not an exact copy of these cattle, but were something unique, similar to a special breed formed under the influence of local conditions.”* The first studbook of the Tagil breed was published in 1931 [18]. Representatives of the Tagil breed of that time are shown in Figure 6a,b. In 1990, the number of Tagil cattle was 148.4 thousand animals [64]. Since the late 1990s, in order to increase milk productivity, accumulation cross breeding of Tagil cattle with Holstein bulls has been practiced. As a result, the number of purebred Tagil cattle has dramatically decreased and in 2023 amounted to only 120 animals, including 64 cows with milk productivity records, that are maintained in a single farm in the Urals [30]. The conducted studies of modern representatives of the Tagil breed in comparison with other breeds of black pied cattle and the Holstein breed using 112,526 SNPs showed the presence of animals with a minimal admixture share relative to the Holstein and other breeds of black pied cattle [65]. Such animals were used as donors for oocyte retrieval via OPU [28] (Figure 6c). The retrieved oocytes were employed to obtain in vitro embryos using semen specimens from Tagil bulls born in 1971 and 1986 that were stored in a cryobank for 35–50 years. As a result, 62 embryos of the Tagil breed from six donor heifers were produced and placed in a cryobank for long-term storage. The viability of these embryos was confirmed by producing offspring after transfer of both fresh and frozen embryos (Figure 6d). In addition, as a result of AI of donor heifers, four bulls were produced, from two of which 1200 doses of semen were supplied to the long-term storage. Thus, owing to this effort, the Tagil breed risk status was lowered from *critical* to *critical-maintained* [28,66,67,68,69].

#### 5.3.2. Kostroma Cattle Breed

**Figure 7:**
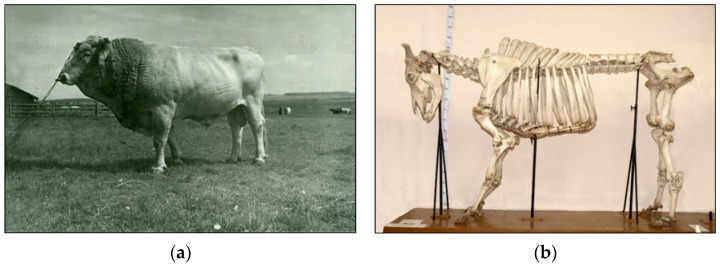

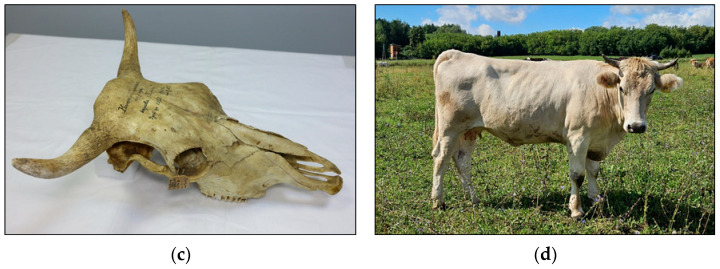
Specimens used for tracing the Kostroma breed specific genetic components: (**a**) Bull Salat 1216, Kostroma breed founder (born in 1942, body weight of 852 kg, breed champion at the 1954 All-Russian Trade Exhibition) [72]; (**b**) bull Salat 1216 skeleton of the Kostroma breed, Museum of the Kostroma State Agricultural Academy; (**c**) Kostroma cattle skull, E.F. Liskun Museum of the Animal Husbandry, Moscow Timiryazev Agricultural Academy (inscription on the scull: of Kostroma breed; cow Proza; milk yield in 1952, 6300 kg; fat, 3.8%); (**d**) donor heifer of the Kostroma breed that carried a large part of authentic genomic components [28].

Another implementation illustration of the key conservation strategy for AnGR is the Kostroma breed. It was created in 1943 as a result of improving local cattle in Central Russia and Belarus by mating with Brown Swiss breeding bulls followed by breeding crossbreeds *inter se* and selecting animals based on milk yield and milk quality [31]. This breed is known for the good suitability of its milk for cheese production. In the USSR, there were 248.3 thousand animals of purebred Kostroma cattle in 1990 [64]. In 2024, the breeding stock of Kostroma cattle declined to 6.59 thousand animals, including 4.14 thousand cows [30]. In the last two decades, Brown Swiss breed bulls have been actively used to improve Kostroma cattle, which has led to a significant decrease in the genomic component proportion of this breed [70]. In this regard, a need arose to identify animals that have retained the largest part of Kostroma cattle genomic component. To obtain the reference DNA profile of the Kostroma breed, bone tissue samples were used from the founder bull Salat 1216 born in 1942 (Figure 7a), whose skeleton is preserved in the Museum of the Kostroma State Agricultural University (Figure 7b), as well as cows of the Kostroma breed from the 1950s–1960s, whose skulls are available in the craniological collection of the E.F. Liskun Museum of Animal Husbandry [71] (Figure 7c).

**Figure 8:**
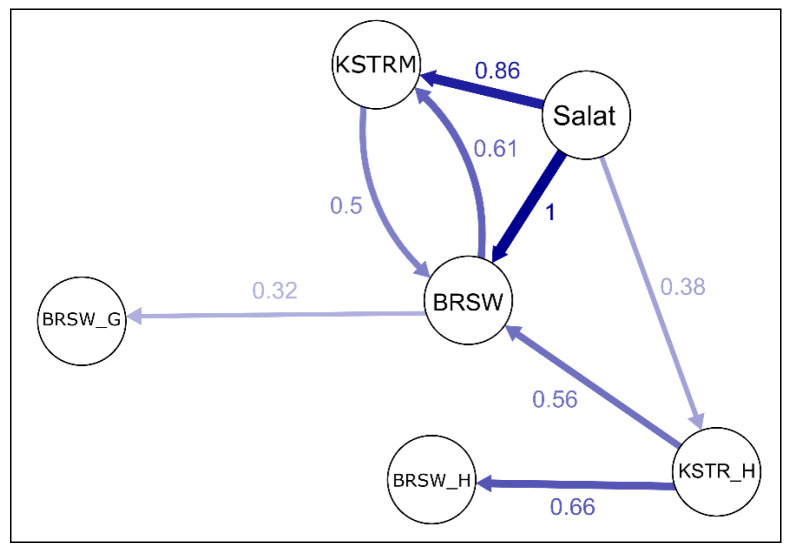
Relative gene migration between the studied populations calculated based on D’Jost estimates using the divMigrate model [60]. Population samples are represented by nodes in the networks: Salat, founder of the Kostroma breed bull Salat 1216; KSTRM_H (*n* = 3) and BRSW_H (*n* = 5), craniological collection skulls of historical Kostroma and Brown Swiss cattle, respectively, dated back to 1950s, from the E.F. Liskun Museum of the Animal Husbandry, Moscow Timiryazev Agricultural Academy; KSTRM (*n* = 60) and BRSW (*n* = 23), modern representatives of Kostroma and Brown Swiss cattle of Russian origin; BRSW_G (*n* = 57), modern Brown Swiss cattle of German origin. Connecting arrows between two nodes represent the direction of gene flow between pair of populations. The digits near the connecting arrows and the arrow color reflect the relative strength of gene flow.

Comparative studies of contemporary Kostroma breed representatives using DNA chips showed the introgression of genes from Salat 1216 into the modern Kostroma breed (Figure 8) [60] and also made it possible to identify animals carrying the largest proportion of ancestral genomic components. Such animals were selected as donors (Figure 7d) for generating embryos (using in vitro embryo production and Ovum Pick-Up techniques) and storing them in the biobank.

#### 5.3.3. Orenburg Goats

**Figure 9:**
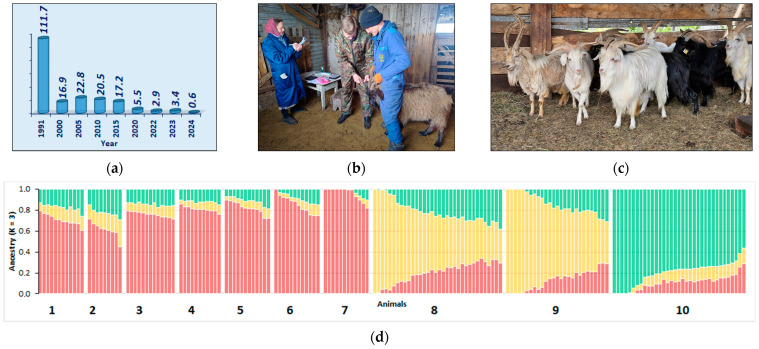
Implementing the strategy for the conservation of Orenburg goats [28,75]: (**a**) Dynamics of changes in the number of Orenburg goats from 1991 to 2024 (in thousand animals). (**b**) Sampling of Orenburg goats as part of an expedition to Orenburg Oblast in 2024. (**c**) The most typical animals selected for the creation of a cryobank of semen and embryos. (**d**) Genetic relationships between modern and archival representatives of the Orenburg breed: 1–4, archival samples from gene pool farms: 1, gray variety (2012); 2, white variety (2017); 3, gray variety (2017); 4, gray variety (2019); 5–7, samples collected at private farms during expedition in 2024: 5, gray variety (private farm 1); 6, gray variety (private farm 2); 7 (private farm 2); 8–10, Russian native mohair breeds used for comparison: 8, Altai Mountain; 9, Altai White; 10, Soviet Mohair. Individual ancestry proportions in the populations under study are displayed in various colors in admixture bar plots [75].

A case of the effective materialization of the strategy in other farm animal species is the preservation of the landmark native breed of Orenburg goats. This breed, created in the 18th century in the Orenburg province using folk selection, was world famous for the high quality and beautiful color of its down fibers [73,74,75,76]. Warm openwork shawls were handmade from the down of these goats and received prizes at world exhibitions. The first gene pool farm was created in 1932 [73,76] and the last one was closed in 2023. Due to changes in demand for the down hair of Orenburg goats [77], a critical decline in the numbers of this breed has been noted. In particular, over the last 40 years, the number of goats has decreased from 111.7 thousand to 0.6 thousand [33,78,79] (Figure 9a). Microsatellite marker profiles [80] and whole-genome SNP genotypes were generated for goat samples collected during expeditions to Orenburg Oblast in 2024 (Figure 9b) and archival samples collected from gene pool farms dating back to 2012, 2017 and 2019. Comparative genomic analysis showed that modern populations have retained authentic genomic components characteristic of the “classic” representatives of the Orenburg breed [75] (Figure 9c). This pilot study allowed the selection of the most representative animals to establish a cryobank of semen and embryos to preserve the breed (Figure 9d), which is instrumental for reducing the breed risk status from *vulnerable* to *vulnerable-maintained* [28].

### 5.4. Genetic Monitoring of Russian AnGR

#### 5.4.1. Whole-Genome Resequencing

The availability of information on DNA profiles of deposited samples using various molecular genetic tools, such as WGS whole-genome genotyping using DNA microarrays, microsatellite marker analysis and studies of individual polymorphisms associated with economically important phenotypes, contributes to the increased demand for bioresource collections [7,56,63,75,80,81]. The most preferred method is WGS data generation, since it allows one to assess the entire spectrum of genetic variability. At present, out of 35 farm animal species subject to breeding in the Russian Federation, reference genome assemblies at the chromosome level are available for 30 species (except for Altai wapiti, common raccoon dog, ferret, nutria and long-tailed chinchilla). At the L.K. Ernst Federal Research Center for Animal Husbandry, WGS data were produced for approximately one thousand animals of various species [82] (Table 3).

The use of WGS technology made it possible to characterize the genomic architecture of native animal breeds and detect genomic regions subject to selective pressure in the course of long-term artificial selection. For instance, patterns of runs of homozygosity (ROH) in the genome of two native goat breeds, Orenburg and Karachay, were studied. Analysis of ROH islands demonstrated their enrichment in the Karachay breed with genes primarily affecting immunity and milk productivity and in the Orenburg breed with genes associated with reproductive qualities and growth rate [115]. Analysis of the sequences of the main reproductive genes in sheep (*GDF9, BMP15* and *BMP15B*) retrieved from WGS data made it possible to identify new polymorphisms in the Romanov, Tushin and Karachay sheep breeds [116]. Complete genomes of museum specimens of the Livny pig breed were used to develop reference DNA profiles of this breed, which can be used to select animals for inclusion in ex situ conservation programs [57]. Analysis of WGS data enabled to identify selective footprints in the genomes of the oldest native chicken breeds Ushanka and Orloff Mille Fleur, as well as more recent Russian White [117,118,119]. The increased availability of WGS technology, due to reduced sequencing costs, as well as the steady growth in the number of species for which reference genomes have been gained, suggest both an expansion of the range of farm animals whose genomes have been sequenced to the chromosome level and the quantity of sequenced samples.

#### 5.4.2. SNP Genotyping Arrays

A highly informative tool for generating the farm animal genomic data is the use of DNA chips, which allow genome-wide genotyping by tens and even hundreds of thousands of SNPs evenly distributed throughout the genome. Currently, DNA microarrays are commercially available for at least ten farm animal species (Table 4). The results of whole-genome genotyping using DNA chips are instrumental for the genetic identification of samples placed in storage, creation of breeds’ references for selection of the most typical representatives for conservation programs, study of the dynamics of allele pool variability, determination of genome-wide selective trajectories and genome-wide association studies (GWAS). At the L.K. Ernst Federal Research Center for Animal Husbandry, genotypes were produced for 12,095 samples of five farm animal species deposited in the bioresource collection, including cattle, pig, sheep, goat and chicken (Table 4) [82].

In particular, using the Bovine SNP50 BeadChip (Illumina, San Diego, CA, USA), the demographic history of five Russian cattle breeds (Bestuzhev, Kholmogory, Kostroma, Red Gorbatov and Yaroslavl) was clarified [124]. Subsequently, the sample of cattle breeds used for the analysis was expanded by including the Yakutian, Suksun, Kalmyk and Russian Black Pied breeds [125]. The Yakutian and Suksun breeds are classified as *endangered* (Supplementary Table S1) and require active conservation programs to save them from extinction. The Kalmyk breed is the main beef breed used in the southern steppe regions of Russia [7]. These are very hardy, medium-sized animals capable of surviving in poor forage conditions and quickly gaining weight with improved feeding [7,18]. Studying the genetic architecture of this breed is essential for developing effective breeding programs. The Russian Black Pied cattle breed has been intensively crossed with Holsteins over the past three decades, making the selection of animals without visible admixture of foreign genomic components valuable for population conservation.

Application of three bioinformatics approaches (computation of *F*_ST_ values, detection of ROH islands and hapFLK method) to analyze whole-genome SNP genotyping data generated using the high-density (HD) DNA microarray Bovine HD BeadChip (Illumina) aided in identifying genomic regions under selection pressure in two oldest native cattle breeds, Kholmogory and Yaroslavl. Hereby, both known and new structural candidate genes were identified [126]. Using the Bovine GGP HD BeadChip (Illumina), the conservation of a significant proportion of authentic genomic components in five Russian dairy breeds of black pied cattle (Russian Black Pied, Istobensk, Kholmogory, Tagil and Yaroslavl) was demonstrated. Accordingly, individuals that had no visible admixture with Holstein cattle were identified and serve as priority animals for inclusion in ex situ conservation programs [65].

Based on 50K SNP data, Deniskova et al. [127] performed the first genome-wide survey of 25 Russian sheep breeds. In that study, the effective population size was calculated and the differentiation between native breeds belonging to different wool types was examined. Subsequently, using the SNP genotypes of world sheep breeds, new knowledge was attained in respect to the origin and evolution of native breeds, which was supplemented by novel data in the implementation context of international cooperation projects. For example, the relationships between fat-tailed coarse wool breeds of Russia and sheep breeds from Kyrgyzstan, through which migration routes passed, were explored [128]. SNP genotypes of native fine wool breeds were integrated into the study of the worldwide pool of Merino sheep breeds [129] and used to elucidate the structure of the Gotland sheep population, in the creation of which they participated [130]. Based on SNP genotyping data using the Ovine Infinium^®^ HD SNP BeadChip (Illumina), a search was conducted for genomic regions under selection pressure in 15 Russian sheep breeds. Genes associated with both adaptation to various climatic conditions, economically important traits (wool, meat and milk performance and reproductive qualities) and exterior features were found [131]. Copy number variation (CNV) analysis in these breeds demonstrated that genes overlapping with CNVs are associated with domestication, adaptation, reproduction, lipid metabolism and resistance to parasitic diseases [132]. The GWAS outcome based on SNP genotypes using the Ovine Infinium^®^ HD SNP BeadChip (Illumina) and F_2_ sheep from a resource population generated by crossing Katahdin rams and Romanov ewes made it possible to identify SNPs associated with body weight at different ages [133].

The Goat SNP50 BeadChip (Illumina) was effective when addressing genetic diversity and characterizing ROH patterns of goat breeds raised in Russia [134]. Furthermore, Neighbor-Net analysis and local and global breeds’ clustering showed that Turkish and Russian local breeds share a close genetic relationship. Based on the analysis of 50 K genotypes, genes located in the ROH islands of the Altai Mountain, Soviet Mohair, Dagestan Down-hair and Altai White Down-hair breeds were established that were associated with lactogenesis, immunity, fineness of down fibers, regulation of reproductive qualities, lipid metabolism and muscle development [135]. In addition, SNP genotypes of Saanen goats maintained in Russia were utilized for exploration of selective sweeps as a response to environmental adaptation in four transboundary goat breeds [136]. Sermyagin et al. [137] carried out a GWAS in a specially developed backcross resource population produced by crossing Kalahari Red and Karachay goats. As a result, genes included in quantitative trait loci (QTLs) and associated with development and growth parameters (osteogenesis and myogenesis), embryogenesis, feed efficiency and meat productivity were identified.

By means of the Porcine SNP60 BeadChip (Illumina), Traspov et al. [138] performed the first genomic survey of 170 pigs representing 13 breeds from the former Soviet Union countries, including Russia.

Using the BovineHD BeadChip, Kharzinova et al. [139,140] studied genetic diversity and addressed genetic relations between five reindeer breeds inhabited different climatic zones of Russia. The contrasting patterns in the genetic structure of the tundra and taiga reindeer were found according to their morphological and ecological differences.

DNA microarrays have also been widely used to characterize local chicken breeds maintained in the in vivo collection at the RRIFAGB–L.K. Ernst Federal Research Center for Animal Husbandry Branch. Dementieva et al. [141] genotyped representatives of 14 small breeds using the Chicken 60K SNP BeadChip (Illumina) to identify the most purebred individuals using homozygosity patterns. The genomic architecture underlying the formation of economically important traits in the Russian White breed was assessed [142,143,144,145]. In the ROH islets of the Russian White population, genes associated with lipid metabolism (*SOCS3*, *NDUFA4*, *TXNRD2*, *IGFBP1* and *IGFBP3*), maintaining body temperature in cold environments (*ADIPOQ, GCGR* and *TRPM2*), non-shivering thermogenesis (*RYR2, CAMK2G* and *STK25*) and muscle development (*METTL21C*) were identified [146]. Genome-wide SNP genotyping revealed genes associated with cold adaptation in local chicken breeds [117,118].

#### 5.4.3. Microsatellite Markers

Even though WGS or SNP chips are increasingly being used for genome research, microsatellite markers are still a potent tool for determining population trends in non-human systems [147,148,149], such as cattle [150]. Moreover, microsatellites are the gold standard for parentage testing in most cattle breeding programs [151]. In relation to samples stored in bioresource collections, employment of microsatellites has also not lost its relevance. Microsatellite analysis provides information on the kinship of individuals, which allows excluding duplicates, as well as selecting the most unrelated animals for studies using DNA microarrays or WGS [56,152]. In addition, in the course of the long-term use of microsatellites for molecular genetic studies in farm animals, a relatively large number of samples from modern representatives of local cattle breeds were genotyped, including those breeds for which whole-genome SNP genotypes were unavailable or were obtained only for a limited number of samples [153,154]. Analysis of microsatellite loci is also a technique of choice when investigating species, for which the reference genomes and DNA microarrays are still absent [155]. Using microsatellite marker panels recommended by the International Society of Animal Genetics (ISAG) [149,156,157], as well as custom panels, genotype datasets for more than 237 thousand samples from 13 species of farm animals were generated at the L.K. Ernst Federal Research Center for Animal Husbandry [82] (Table 5).

Microsatellite markers have been actively employed for comparative studies of modern and historical cattle populations in order to study the dynamics of changes in their allele pool in Russian Black Pied cattle breeds [56] and steppe cattle of the former Soviet Union countries [7]. Using microsatellite loci, the genetic diversity and population structure of Dagestan Mountain cattle were assessed for the first time, on the basis of which animals with low levels of admixture were established as potential germplasm donors [158]. Based on the microsatellite markers, large-scale monitoring of the genetic diversity in local (Kemerovo, Livny and Tsivilsk) and transboundary (Duroc, Landrace, Large White, Berkshire and Mangalica) pigs was carried out [140]. The population structure of domestic reindeer was investigated, samples of which were collected in all areas of its habitat in Russia, i.e., from the Kola Peninsula in the west to Chukotka in the east [159,160].

#### 5.4.4. New Prospects for Less Studied Species: A Case of Goose

**Figure 10:**
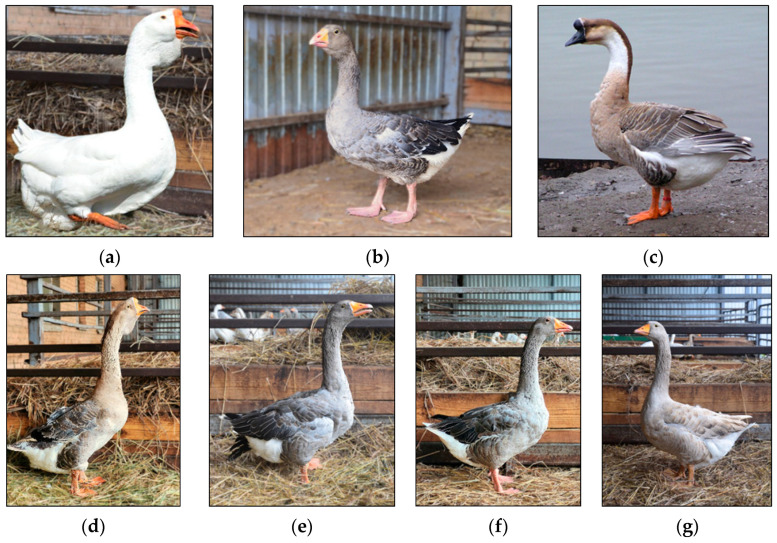
Goose breeds selected for in vitro conservation at the L.K. Ernst Federal Research Center for Animal Husbandry. (**a**) Kholmogory, (**b**) Tula Fighting, (**c**) Chinese Gray, (**d**) Kholmogory Gray, (**e**) Large Gray, (**f**) Shadrinsk, (**g**) Vladimir Clay. Credit: (**a**,**b**,**d**–**g**), own photos; (**c**), https://commons.wikimedia.org/wiki/File:Anser_cygnoides_dom_Hoeckergans_2.jpg (accessed on 27 August 2025; CC BY-SA 3.0).

Within the framework of the project “Development of a Network of Bioresource Collections of Farm Animals” supported by the Ministry of Education and Science of the Russian Federation [82], measures have been planned for genetic monitoring of AnGR preserved ex situ using various molecular genetic tools (microsatellite analysis and whole-genome genotyping based on DNA microarrays and WGS) to develop biodiversity conservation strategies. This research program targets breeds of sheep and horses preserved in in vitro collections, as well as breeds of geese, turkeys and silkworm preserved in in vivo animal collections. For instance, goose farming is traditional in Russia [161], with 28 goose breeds of both native and foreign origin being presently officially recognized and approved for selective breeding [26]. However, the numbers of many of these remarkable gene pool breeds have declined sharply and some are on the verge of extinction. This makes genetic diversity and population structure studies crucial for developing efficient conservation and selective breeding programs to preserve goose breed biodiversity [162]. The only gene pool collection for 21 goose breeds in Russia is maintained at the Upper Volga Federal Agricultural Scientific Center [163,164], although molecular genetic studies of these breeds have not been undertaken previously. In order to characterize the biodiversity and select animals for inclusion in semen cryopreservation programs, the respective samples were collected to carry out a preliminary study [165] of the genetic diversity of seven Russian goose breeds maintained in the gene pool collection. It included the Chinese Gray, Tula Fighting, Shadrinsk, Large Gray, Vladimir Clay, Kholmogory and Kholmogory Gray (Figure 10, Table 6) that were genetically assessed using eleven microsatellite markers. Also, the genetic relationships among them were examined to reconstruct their history, providing valuable insights for future conservation and sustainable management programs [165]. For these purposes, the corresponding statistical analyses were performed using such R package (version 4.0) components as *diveRsity* (version 1.9.89) [166], *adegenet* (version 2.1.11) [167], *ggplot2* (version 4.0.0) [168] and *pophelper* (version 2.3.1) [169], and software programs Structure (version 2.3.4) [170] and SplitsTree (version 4.14.6) [171] that are usually employed when analyzing genetic structure and diversity in other farm animals.

As a result, the phylogeny pattern established for seven breeds using microsatellite markers coincides with early phenetic data [175,176,177] and with the literature data on the origin of breeds. The Chinese and Kholmogory (of two varieties) breeds originated from the Chinese root (i.e., an ancestral wild species—swan goose *Anser cygnoides*). The Large Gray and Shadrinsk breeds descended from the European root (i.e., an ancestral wild species—graylag goose *A. anser*). The Vladimir Clay breed, both according to phenetic calculations and molecular genetic data, occupies a separate, independent phylogenetic branch. Herewith, the Tula Fighting breed also represents a separate central node relative to other breeds [165]. The results of the diversity analysis of seven Russian goose breeds from a genebank collection using microsatellite markers [165] are presented in Table 7.

Two goose varieties of Kholmogory origin (KHLM and KHLM_GR) exhibited the greatest allelic richness (*A*_R_) values (3.215 ± 0.269 and 3.236 ± 0.341, respectively). Notably, these two breeds differed from the remaining ones by the maximum genetic diversity values of the unbiased expected heterozygosity (_U_*H*_E_ = 0.492 ± 0.078 and 0.504 ± 0.063, respectively), revealing robust allelic and genetic diversity. A comparably high value of genetic diversity was also observed in the Chinese Gray breed (*A*_R_ = 2.913 ± 0.352 and _U_*H*_E_ = 0.492 ± 0.079). In contrast, the Vladimir Clay showed the lowest value of the indices (*A*_R_ = 2.064 ± 0.215 and _U_*H*_E_ = 0.299 ± 0.066), suggesting a reduced genetic variability. The inbreeding coefficient (_U_*F*_IS_), ranging from −0.032 to 0.039, revealed that five breeds were characterized by the minimum level of extinction risk. An exception was noted for Tula Fighting and Vladimir Clay geese, in which the inbreeding coefficient estimates were fairly positive (_U_*F*_IS_ = 0.138 and 0.086, respectively). However, the broad confidence interval [−0.085 to 0.361] and [−0.084; 0.256] indicated statistical insignificance (*p* > 0.05) [165]. To the best of our knowledge, this is the first attempt to gain insight into genetic diversity of the goose gene pool in Russia using molecular genetic markers. Although based on a limited sample size, these findings underscore the vital need to monitor genetic diversity in the native goose breeds that harbor unique characteristics. Based on the found microsatellite marker genotypes, the individuals were selected for both germplasm cryobanking and WGS [165]. Maintaining the genetic diversity, along with enhancing performance, remains a fundamental challenge for native breeds [178,179,180,181]. In prospect, the efforts like this will provide the foundation for sustainable use, development and conservation of Russian poultry genetic resources that occupy a peculiar position in the diversity of Eurasian poultry breeds [179,182,183,184,185].

## 6. Conclusions

In Russia, as in other countries, the livestock sector utilizes a limited number of highly productive breeds, and native breeds have been subject to genetic erosion and degradation in recent decades. For example, from 1990 to 2024, the Orenburg goat population declined from 111,700 to 600 animals; the Tagil cattle population declined from 148,400 to 110 animals; and the Kostroma cattle population declined from 248,300 to 6590. It should be noted that positive trends have emerged in recent years in terms of centralized support measures and appropriate management to preserve the genetic diversity of native breeds and livestock populations. These include the establishment of the National Center for AnGR, network expansion and grant support for bioresource collections, along with the modernization, inventory and replenishment of cryobanks of preserved genetic material suitable for reproduction. In particular, the L.K. Ernst Federal Research Center for Animal Husbandry is actively developing scientifically sound strategies for preserving local agricultural AnGR. These strategies include genetic monitoring of farm animal populations using the latest molecular genetic approaches, the creation of breed genomic standards based on museum and archival specimens and the selection of donors to obtain gametes and embryos for long-term storage in cryobanks, as well as for sale to AI companies. This review presents examples of the effective, full or partial implementation of these strategies (the Tagil and Kostroma cattle breeds and the Orenburg goat breed). As part of the new program for the development of bioresource collections, research based on various molecular genetic approaches is planned for the genetic monitoring of previously unused AnGR currently conserved ex situ (geese, turkeys and silkworms). In summary, active work is currently underway to promote and implement the objectives outlined in the UN Convention on Biological Diversity relative to the biodiversity conservation of agricultural animals. We hope that the listed activities and measures implemented in Russia will contribute to the conservation of global biodiversity by preserving local genetic resources adapted to a wide range of natural and climatic conditions and ecogeographic zones. This will create additional preconditions for the sustainable development of livestock farming in a changing climate.

## Figures and Tables

**Table 1:** The number of registered breeds, intra-breed types and line crosses by animal species permitted for breeding in Russia [26].

#	Animal Species		Number		
			Breeds *	Types	Crosses
1.	Altai wapiti	*Cervus canadensis sibiricus*	2 (2 **)	2	
2.	American bison	*Bison bison*	1		
3.	American mink	*Neogale vison*	17	10	
4.	Arctic fox	*Vulpes lagopus*	2 (2)	3	
5.	Bactrian camel	*Camelus bactrianus*	2 (1)	1	
6.	Cattle	*Bos taurus*	80 (25)	55	
7.	Chicken	*Gallus gallus*	79 (32)		100
8.	Common ostrich	*Struthio camelus*	1		
9.	Common raccoon dog	*Nyctereutes procyonoides*	1 (1 **)		
10.	Domestic duck	*Anas platyrhynchos*	3 (1)		10
11.	Domestic guinea fowl	*Numida meleagris*	5 (5)		
12.	Donkey	*Equus asinus*	1 (1 **)		
13.	European rabbit	*Oryctolagus cuniculus*	13 (9)		4
14.	Ferret	*Mustela furo*	3 (3)		
15.	Garden snail	*Cornu aspersum*	2		
16.	Goat	*Capra hircus*	35 (12)	3	
17.	Graylag goose	*Anser anser*	28 (17)		1
18.	Horse	*Equus caballus*	70 (33)	7	
19.	Japanese quail	*Coturnix japonica*	13 (3)		1
20.	Long-tailed chinchilla	*Chinchilla lanigera*	1 (1)		
21.	Muscovy duck	*Cairina moschata*	1 (1 **)		3
22.	Nutria	*Myocastor coypus*	1	2	
23.	Pig	*Sus domesticus*	27 (10)	15	
24.	Red fox	*Vulpes vulpes*	4 (3)	8	
25.	Reindeer	*Rangifer tarandus*	4 (4)	1	
26.	Roman snail	*Helix pomatia*	2 (2 **)		
27.	Sable	*Martes zibellina*	2 (2)	1	
28.	Sheep	*Ovis aries*	77 (41)	24	
29.	Sika deer	*Cervus nippon*	2 (2 **)		
30.	Silkworm	*Bombyx mori*	9 (9)		12
31.	Turkey	*Meleagris gallopavo*	7 (6)		10
32.	Water buffalo	*Bubalus arnee (bubalis)*	1 (1)		
33.	Western honeybee	*Apis mellifera*	7 (6)	7	
34.	Yak	*Bos grunniens*	2 (1)	1	
35.	Zebu	*Bos indicus*	2		
	Total		507 (236)	140	141

* Breeds, total number (number of native breeds, including ** domesticated forms).

**Table 2:** Local cattle breeds preserved in gene pool farms and their present population size [30,37,38].

#	Breed	Population Size								
		Total, Thousand Animals			Cows, Thousand Animals			Bulls, Animals		
	Year of Recording	2022	2023	2024	2022	2023	2024	2022	2023	2024
1.	Bestuzhev	0.72	–	–	0.45	–	–	–	–	–
2.	Brown Caucasian	2.02	0.67	1.46	1.22	0.34	1.13	–	–	4
3.	Dagestan Mountain	0.64	0.27	0.18	0.39	0.22	0.11	3	3	2
4.	Istobensk	0.73	0.75	0.74	0.46	0.46	0.46	–	–	–
5.	Pechora type ^1^	0.30	0.18	0.19	0.29	0.14	0.14	–	–	–
6.	Red Gorbatov	–	0.28	–	–	0.21	–	–	–	–
7.	Red Steppe	1.76	0.68	0.77	1.04	0.42	0.42	–	–	–
8.	Sychyovka	–	–	0.32	–	–	0.18	–	–	–
9.	Tagil	0.14	0.12	0.11	0.09	0.08	0.08	–	–	–
10.	Yakut	0.83	0.70	0.96	0.35	0.32	0.39	24	24	28
Total		7.14	3.74	4.72	4.29	2.18	2.90	27	27	34

^1^ Most northern population of Kholmogory cattle.

**Table 3:** Genomes of farm animal species sequenced to chromosome level ^1,2^.

#	Animal Species	No. of Chromosomes (2*n*)	Size, in Gb	Year	References	*n*
1.	Chicken (*Gallus gallus*)	78	1.05	2004	[83]	183
2.	European rabbit (*Oryctolagus cuniculus*)	44	2.67	2005	[84]	–
3.	Western honeybee (*Apis mellifera*)	16	0.23	2006	[85]	–
4.	Cattle (*Bos taurus*)	60	2.91	2009	[86,87]	269
5.	Horse (*Equus caballus*)	64	2.47	2009	[88]	–
6.	Pig (*Sus scrofa*)	38	2.20	2009	[89]	47
7.	Sheep (*Ovis aries*)	54	2.71	2009	[90]	204
8.	Turkey (*Meleagris gallopavo*)	80	1.08	2009	[91]	10
9.	Yak (*Bos grunniens*)	60	2.8	2012	[92]	12
10.	Domestic duck (*Anas platyrhynchos*)	80	1.21	2013	[93]	–
11.	Goat (*Capra hircus*)	60	2.92	2013	[94]	83
12.	American bison *(Bison bison)*	60	2.8 (3.0)	2014	[95]	–
13.	Swan goose *(Anser cygnoides)*	80	1.12	2015	[96]	60
14.	Japanese quail (*Coturnix japonica)*	31	0.93	2015	[97]	–
15.	Domestic guinea fowl (*Numida meleagris*)	31	1	2017	[98]	–
16.	Reindeer (*Rangifer tarandus*)	70	2.64	2017	[99]	96
17.	Silkworm (*Bombyx mori*)	28	0.460	2018	[100]	–
18.	Roman snail (*Helix pomatia*)	54	–	2019	[101]	–
19.	Donkey (*Equus asinus*)	31	2.79	2020	[102]	–
20.	Arctic fox (*Vulpes lagopus*)	25	2.35	2021	[103]	–
21.	Muscovy duck (*Cairina moschata*)	39	1.1	2021	[104]	–
22.	Water buffalo (*Bubalus arnee (bubalis)*)	50	2.6	2022	[105]	–
23.	American mink (*Neogale vison*)	15	2.68	2022	[106]	–
24.	Zebu *(Bos indicus)*	60	2.7	2023	[107]	–
25.	Sika deer (*Cervus nippon)*	34	2.78	2024	[108]	–
26.	Common ostrich (*Struthio camelus*)	41	1.5	2024	[109]	–
27.	Garden snail (*Cornu aspersum*)	27	2.91	2024	[110]	–
28.	Red fox (*Vulpes vulpes*)	17	2.4	2025	[111]	–
29.	Sable (*Martes zibellina*)	19	2.39	2025	[112]	–
30.	Bactrian camel (*Camelus bactrianus*)	37	2.5	2025	[113]	–

^1^ According to Eggen [114] with additions. ^2^ Animal species are listed in the order of publication of the first genome assembled to the chromosome level; genome size (in Gb, billion base pairs) is listed in accordance with the size of the assembly; year is the year of publication of the first genome assembly for the species to the chromosome level; *n* is the number of complete genomes sequenced at the L.K. Ernst Federal Research Center for Animal Husbandry.

**Table 4:** The most commonly used commercially available microarrays for genome-wide SNP screening of farm animals.

Animal Species	Manufacturer	DNA Chip Name	No. of SNPs	*n* ^1^
Cattle	Illumina	Bovine HD BeadChip	>777,000	329
	Affymetrix (Santa Clara, CA, USA)	Axiom^®^ Genome-Wide BOS1	>640,000	–
	Illumina	Bovine GGP HD BeadChip	~150,000	1078
	Affymetrix	Axiom Bovine 100K Array	~100,000	–
	Affymetrix	Axiom Bovine Genotyping v3	63,988	–
	Illumina	Bovine SNP50 v3 BeadChip	53,714	1385
	Illumina	Bovine LD v2 BeadChip	7931	–
Pig	Affymetrix	Axiom^®^ Porcine Array	658,692	–
	Illumina	Porcine GGP BeadChip	~80,000	1313
	Illumina	Porcine SNP50 v2 BeadChip [120]	64,232	4400
	Affymetrix	Axiom Porcine Breeders Genotyping Array	55,232	–
Sheep	Illumina	Ovine Infinium^®^ HD SNP BeadChip [121]	603,350	864
	Illumina	Ovine SNP50 BeadChip	54,241	1530
	Affymetrix	Axiom Ovine Genotyping Array (51K)	~51,000	–
Goat	Affymetrix	Axiom Goat Genotyping v2	59,795	–
	Affymetrix	Axiom Goat Genotyping v1	58,655	–
	Illumina	Goat SNP50 BeadChip [122]	52,295	668
Horse	Affymetrix	Axiom^®^ Equine Array	670,796	–
	Illumina	GGP Equine BeadChip	~70,000	–
	Illumina	Equine 50K BeadChip	~50,000	–
Buffalo	Affymetrix	Axiom^®^ Buffalo Array	90,000	–
Reindeer	Affymetrix	OVSNP600	702,183	–
	Affymetrix	OVSNP60	72,723	–
Camelids	Affymetrix	Axiom Camelids Genotyping	59,938 ^2^	–
Chicken	Affymetrix	Axiom^®^ Genome-Wide Chicken Array	>580,000	–
	Illumina	Chicken SNP BeadChip [123]	57,636	528
Turkey	Affymetrix	Axiom Turkey Genotyping	643,845	–

^1^ *n*, number of samples stored in the bioresource collection for which SNP genotypes were generated at the L.K. Ernst Federal Research Center for Animal Husbandry; ^2^ number of SNPs for *Camelus bactrianus*.

**Table 5:** Number of samples assessed using microsatellite markers in the bioresource collection at the L.K. Ernst Federal Research Center for Animal Husbandry [82].

Animal Species		ISAG ^1^	Custom Panels ^2^	No. of Loci ^3^	*n* ^4^
Cattle	*Bos taurus*	+		12	117,230
Pig	*Sus domesticus*	+		10	96,675
Sheep	*Ovis aries*	+		12	11,068
Reindeer	*Rangifer tarandus*		+	9	4929
Goat	*Capra hircus*	+		14	2130
Western honeybee	*Apis mellifera*		+	7	1921
Chicken	*Gallus gallus*	+		25	1527
Yak	*Bos grunniens*		+	11	850
Turkey	*Meleagris gallopavo*		+	6	436
Goose	*Anser anser*/*A. cygnoides*		+	11	149
Altai wapiti	*Cervus canadensis sibiricus*		+	14	126
American bison	*Bison bison*		+	15	94
Bactrian camel	*Camelus bactrianus*		+	17	46

^1^ ISAG, availability (+) of panels recommended by the International Society of Animal Genetics (ISAG) [149,156,157]. ^2^ Custom panels, availability (+) of custom multiplex microsatellite panels. ^3^ No. of loci, number of microsatellite loci genotyped. ^4^ *n*, number of genotyped samples from the bioresource collection, L.K. Ernst Federal Research Center for Animal Husbandry.

**Table 6:** Goose breeds from the gene pool collection examined with 11 microsatellite markers.

Breed	Description	References
Chinese Gray (CN_GR) (Figure 10c)	Originated in Manchuria, spread across China and reached Europe and Russia in the 1700s. Russian breeders crossed it with local geese to create the Kuban, Pereyaslav, Kholmogory and Linda breeds. Distinguished from standard Chinese geese solely by gray plumage, it was included in the State Register of Breeding Achievements (SRBA) [172] in 1993.	[173,174]
Tula Fighting (TULA_FH) (Figure 10b)	One of Russia’s oldest native breeds, developed through traditional folk selection specifically for goose fighting—a popular pastime in Russia, particularly in Tula Governorate from the 17th–18th centuries onward. Characterized by a sturdy build, remarkable endurance and an aggressive fighting spirit. Included in the SRBA [172] in 1993.	[174]
Shadrinsk (SHAD) (Figure 10f)	An ancient indigenous breed developed between the 17th–19th centuries in Trans-Urals (Shadrinsk, Kurgan Oblast) through traditional folk selection of domesticated wild graylag geese. Remarkable for its hardiness, adaptation to harsh climates and excellent meat qualities. Included in the SRBA [172] in 1993.	[173,174]
Large Gray (LR_GR) (Figure 10e)	A relatively young breed developed in the second half of the 20th century. Selective breeding began in Ukraine in the 1930s. In 1941, the breed development continued in Tambov region through crossing Romny and Toulouse geese. Classified as a dual-purpose meat-and-fat type breed. Included in the SRBA [172] in 1993.	[173,174]
Vladimir Clay (VLAD_CL) (Figure 10g)	Developed in Vladimir Oblast in the 1960s–1980s by crossing Kholmogory (for size and hardiness), Tula Fighting (for robust physique) and Chinese (for enhanced egg production) geese. Included in the SRBA [172] in 1993.	[173,174]
Kholmogory (KHLM) (Figure 10a)	One of the Russia’s oldest and most popular breeds. Developed in the 19th century through crossbreeding of Chinese and Arzamas geese near the town of Kholmogory (Arkhangelsk Governorate). The breed became widely valued across Russia due to targeted selection for cold tolerance and high meat yield. Primarily distributed in Oryol, Bryansk, Voronezh, Kursk and Belgorod Oblasts. Included in the SRBA [172] in 1993 (without color specification).	[173,174]
Kholmogory Gray (KHLM_GR) (Figure 10d)	A variety of the Kholmogory goose that is differentiated solely by its distinctive plumage that mimics wild goose coloration (gray-brown feathers on the back and wings with white ventral sections). All other characteristics remain identical to the standard Kholmogory breed.	[173,174]

**Table 7:** Genetic diversity of seven Russian goose breeds based on microsatellites analysis (11 loci) [165].

Breed ^1^	*n* ^2^	*A*_R_ ^3^ (M ^7^ ± SE ^8^)	*H*_O_ ^4^ (M ± SE)	_U_*H*_E_ ^5^ (M ± SE)	_U_*F*_IS_ ^6^ (M ± SE)
CN_GR	21	2.913 ± 0.352	0.481 ± 0.079	0.492 ± 0.079	0.007 [−0.116; 0.130]
TULA_FH	8	2.727 ± 0.273	0.352 ± 0.067	0.403 ± 0.068	0.138 [−0.085; 0.361]
SHAD	11	2.123 ± 0.209	0.306 ± 0.064	0.322 ± 0.055	0.039 [−0.165; 0.243]
LR_GR	14	2.337 ± 0.214	0.377 ± 0.059	0.371 ± 0.058	−0.032 [−0.15; 0.086]
VLAD_CL	18	2.064 ± 0.215	0.278 ± 0.069	0.299 ± 0.066	0.086 [−0.084; 0.256]
KHLM	14	3.236 ± 0.341	0.481 ± 0.093	0.492 ± 0.078	−0.003 [−0.218; 0.212]
KHLM_GR	14	3.215 ± 0.269	0.506 ± 0.08	0.504 ± 0.063	−0.004 [−0.181; 0.173]

^1^ Breed of geese: CN_GR, Chinese Gray; TULA_FH, Tula Fighting; SHAD, Shadrinsk; LR_GR, Large Gray; VLAD_CL, Vladimir Clay; KHLM, Kholmogory; KHLM_GR, Kholmogory Gray; ^2^ *n*, number of individuals; ^3^ *A*_R_, rarefied allelic richness; ^4^ *H*_O_, observed heterozygosity; ^5^ _U_*H*_E_, unbiased expected heterozygosity; ^6^ _U_*F*_IS_, inbreeding coefficient based on the difference between _U_H_E_ and H_O_ with a 95% confidence interval (CI; in square brackets); ^7^ M, mean value; ^8^ SE, standard error.

## Data Availability

Not applicable.

## Supplementary Materials

The following supporting information can be downloaded at https://www.mdpi.com/article/10.3390/ani15213103/s1. Table S1: Number of controlled livestock subject to selective breeding (2024) and FAO DAD-IS risk categories for dairy and dairy-beef cattle breeds maintained in Russia.

## Abbreviations

The following abbreviations are used in this manuscript:

AI	Artificial insemination
AnGR	Animal genetic resources
DAD-IS	Domestic Animal Diversity Information System
DNA	Deoxyribonucleic acid
ET	Embryo transfer
FAO	Food and Agriculture Organization of the United Nations
SNP(s)	Single nucleotide polymorphism(s)
UN	United Nations
WGS	Whole-genome sequencing
