# Supplementary material for: Conservation of Native Livestock Breeds in Russia: Current State and Promising Prospects
Source: Animals (Basel). 2025 Oct 25;15(21):3103. doi: 10.3390/ani15213103 (PMC12607823; doi:10.3390/ani15213103)
Supplement: Supplementary file 1 [file animals-15-03103-s001.zip › animals-3872373-supplementary.pdf]

Supplementary Table S1. Number [1] of controlled livestock subject to selective breeding (2024) and FAO DAD-IS risk categories for dairy and dairy-beef cattle breeds maintained in Russia.

#	Breed	FAO DAD-IS risk category	Number *		
			Thousand animals		Animals
			Total	Cows	Bulls**
1.	Angeln		0.09	0.05	9 (9)
2.	Ayrshire		59.76	39.63	71 (71)
**3.**	**Bestuzhev**	***at risk***	**6.40**	**3.47**	**21 (3)**
**4.**	**Brown Caucasian**	***endangered***	**1.63**	**1.25**	**4 (–)**
5.	Brown Swiss		15.54	10.09	19 (18)
**6.**	**Dagestan Mountain**	***critical***	**0.18**	**0.11**	**2 (–)**
7.	Danish Red		2.46	1.90	14 (14)
8.	Estonian Red		0.53	0.28	–
9.	Holstein		1807.72	1126.29	1025 (1024)
**10.**	**Istobensk**	***endangered***	**0.75**	**0.46**	**3 (3)**
11.	Jersey		22.02	14.32	21 (21)
12.	Kholmogory	*not at risk*	41.72	25.20	55 (49)
**12a**	**Pechora type**	***critical***	**0.19**	**0.14**	**–**
**13.**	**Kostroma**	***at risk***	**6.59**	**4.14**	**22 (22)**
14.	Montbéliarde		2.91	2.13	6 (6)
**15.**	**Red Gorbatov**	***cryoconserved only***	**–**	**–**	**1 (1)**
16.	Red Pied	*not at risk*	41.73	25.74	24 (24)
17.	Red Steppe	*not at risk*	58.08	33.25	25 (25)
18.	Russian Black Pied	*not at risk*	360.10	202.83	101 (25)
19.	Simmental		68.11	40.74	116 (116)
**20.**	**Suksun**	***endangered***	**1.87**	**1.28**	**1 (1)**
**21.**	**Sychyovka**	***vulnerable***	**3.02**	**1.83**	**2 (2)**
**22.**	**Tagil**	***critical***	**0.11**	**0.08**	**2 (2)**
**23.**	**Yakutian**	***endangered***	**0.96**	**0.39**	**30 (2)**
**24.**	Yaroslavl	*not at risk*	26.87	17.44	18 (18)
	Total		2525.13	1552.87	1592 (1456)

[1] According to Voskresensky et al. [30]. * Number of breeding animals; ** number of sires in breeding enterprises indicated in brackets. Native breeds are highlighted in light blue; the native breeds, which belong to the risk categories, are marked in bold.
